# Effect of vaginal/oral tadalafil on endometrial thickness in IVF patients: a double-blind, placebo controlled RCT: a pilot study

**DOI:** 10.52054/FVVO.14.2.026

**Published:** 2022-07-01

**Authors:** J Balduyck, A Ameye, W Decleer

**Affiliations:** KU Leuven, Resident Obstetrics and Gynaecology, Oude Markt 13, 3000 Leuven, Belgium; Fertility Center, Department of Obstetrics and Gynaecology, AZ Jan Palfijn, Henri Dunantlaan 5, 9000 Ghent, Belgium

**Keywords:** Tadalafil, endometrial thickness, implantation, oral/vaginal application

## Abstract

**Objective:**

To investigate the effect of tadalafil (a long working phosphodiesterase type 5 inhibitor) on the endometrial thickness, biochemical pregnancy rates and clinical pregnancy rates in women in an in vitro fertilization treatment. This study investigates the use of vaginal and oral administration of tadalafil.

**Study design:**

This is a prospective double-blind placebo-controlled randomized controlled trial with 58 patients in an in vitro fertilization treatment with a short antagonist stimulation protocol. The study population is divided into three equal groups comparing oral and vaginal administration of tadalafil to a control group.

**Results:**

No significant difference in endometrial thickness and number of biochemical and clinical pregnancies was found between the three groups.

**Conclusion:**

This study could not show a significant benefit of administration of tadalafil. However, a trend towards more pregnancies in the group treated with oral tadalafil is seen, more research in specific subgroups is needed.

## Introduction

In vitro fertilization (IVF) is a treatment for couples and single women with fertility issues. The existing literature shows that the chance of a pregnancy after a transfer of an embryo depends on, among other things, the thickness of the endometrium. Moreover, this is one of the most important predictors for the chance of a successful implantation and the chance of pregnancy (Benni and Patil, 2016). The chance of implantation increases with the thickness of the endometrium at the time of implantation (Richter et al., 2007). Of course, extremely thick endometrium is no positive sign either, because of tendency to polyp formation or hyperplasia. The preparation of the uterine lining for implantation is, among others (e.g., growth factors and cytokines), dependent on oestrogen and progesterone. These hormones can either be produced by the follicle itself or can be administered exogenously as oestradiol valerate and micronized progesterone (Zegers-Hochschild et al., 2017).

**Figure 1:**
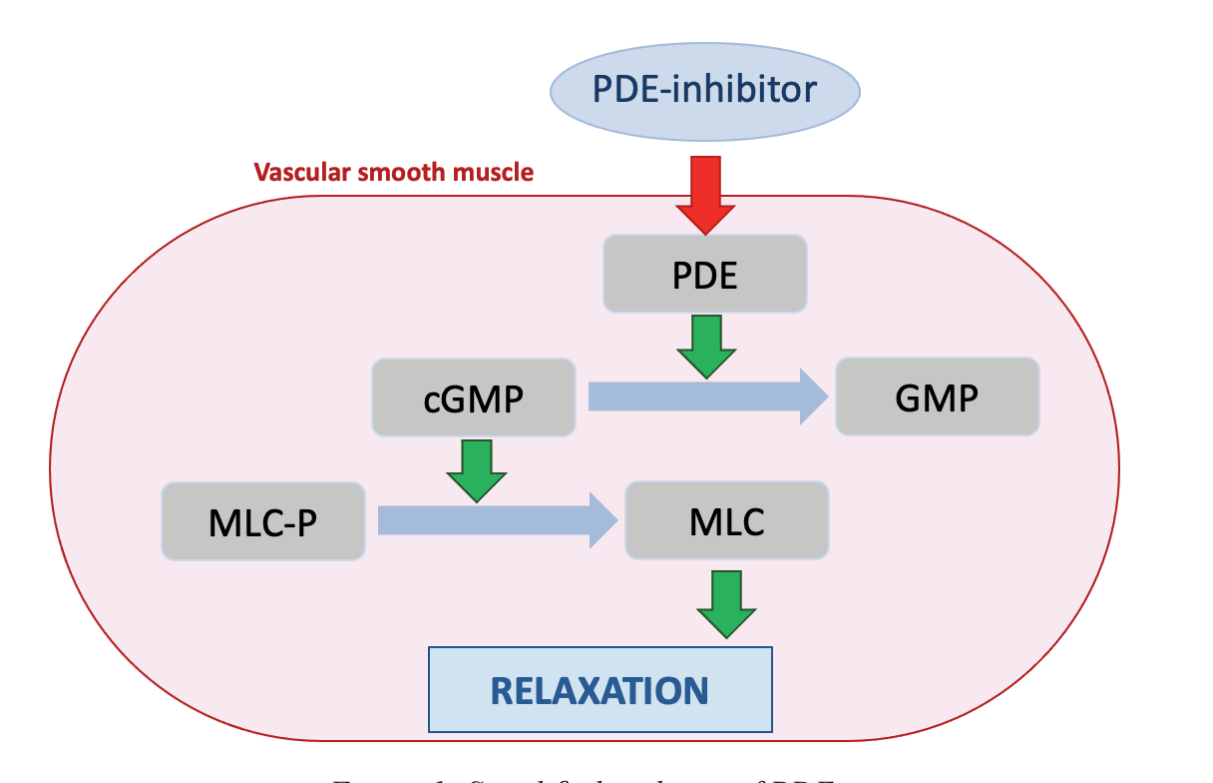
Simplified pathway of PDE. PDE = phosphodiesterase, (c)GMP = (cyclic) guanosine monophosphate, MLC(-P) = myosin light chain (-phosphate).

In addition, a phosphodiesterase type 5 (PDE-5) inhibitor can also be administered (Sher and Fisch, 2000). PDE-5 inhibitors act through the inhibition of phosphodiesterase, resulting in less cyclic guanosine monophosphate (c-GMP) degradation. The accumulation of c-GMP enhances the effect of nitric oxide (NO). As a result, the smooth muscles of the vessels relax, which leads to vasodilation (Figure 1).

PDE-5 inhibitors are used for the treatment of pulmonary hypertension and for erectile disfunction in men. Sher and Fisch (2000, 2002) demonstrated that PDE-5 inhibitors also influence the circulation of the uterine arteries and the development of the uterine lining, partly due to the increased supply of growth factors, cytokines, and hormones.

In previous studies, the effect on the endometrium of sildenafil citrate, a short-acting PDE-5 inhibitor, was investigated (Alieva et al., 2012; Dehghani Firouzabadi et al, 2013; Fahmy et al., 2015; Mishra et al., 2015). Three studies investigated the effect of tadalafil, a long-acting PDE-5 inhibitor, by oral administration (Avilés et al., 2017; Ammar and Salem, 2018; Mendez et al., 2015).

In this study the effect of tadalafil on endometrial thickness, implantation rate and the rate of biochemical and clinical pregnancies is investigated in an IVF treatment. This long-acting PDE-5 inhibitor has a half-life of 17,5 hours (Gupta et al., 2005) and could thus exert a long-term effect on the endometrium resulting in a thicker endometrium and higher implantation rates. In addition, the comparison between oral and vaginal tadalafil administration is investigated, of which there is no literature yet.

The study hypothesis is that treatment with tadalafil provides a significantly better build-up of the uterine lining in the treatment group compared to placebo. Additionally, the secondary hypothesis is that the treatment will have better results vaginally compared to orally.

## Patients and Methods

### Study design and participants

This study is a double-blind, prospective, placebo- controlled randomized controlled trial (RCT). It took place at the AZ Jan Palfijn hospital in Ghent, Belgium. The ethical approval for the project was given by the institutional review board of the AZ Jan Palfijn hospital in Ghent and the OLV hospital in Aalst (reference number: B0126201938559, 25/01/2019).

The study population consists of 60 patients with primary or secondary fertility problems. The size of the study remains limited as this is a pilot study. Following recruitment, patients were divided into three equal groups via computer randomization (1:1:1 ratio) (www.graphpad.com). After the inclusion period, 58 patients were eventually included and divided into 3 equal groups.

The active form contains 10 mg tadalafil. This dose is well below the dose used in pulmonary hypertension (40 mg per day) and equal to the dose used in male genital erectile dysfunction. The placebo form does not contain an active substance.

Group A (n = 20): oral tadalafil and vaginal placebo

Group B (n = 19): oral placebo and vaginal tadalafil

Group C (n = 19): oral placebo and vaginal placebo

Once a day, each group receives the active substance (tadalafil) or placebo, both orally and vaginally. The study medication was bought by the investigators. The local pharmacist converted the oral capsules into vaginal caps and delivered the placebo caps (oral and vaginal).

The patients were included after signing an informed consent in the period between March 2019 and September 2019. Inclusion criteria were a signed informed consent, patients between 18 and 40 years with indication for IVF-treatment, IVF attempt 1 to 3 in the AZ Jan Palfijn fertility centre. Exclusion criteria were BMI > 35 kg/m2, abnormal uterine cavity, known liver, kidney and/ or heart disease, history of myocardial infarction or vascular disease or a contraindication for tadalafil.

Therapy (tadalafil or placebo) started at the beginning of stimulation after control of baseline blood values and stopped on the day of ovulation induction (with a maximum of 12 days). Monitoring of IVF stimulation was carried out according to standard protocol: after the first 6 days of stimulation, follow-up by ultrasound (follicle measurement and measurement of endometrial thickness) and blood sampling was conducted every 2 days. As soon as 3 or more follicles reached a mean diameter of 17mm or more, hCG triggering was administered.

We chose to conduct the study with tadalafil as it has a longer duration of action than sildenafil (Gupta et al., 2005).

Endometrial thickness, the primary outcome, is measured at the day of ovulation induction.

Embryo transfer took place 3 or 5 days after fertilization, based on local guidelines. One or two embryos were transferred according to Belgian law limitations, depending on age and cycle number (Ombelet et al., 2004).

After transfer of the embryo, endometrial support is given by means of micronized progesterone in the form of vaginal caps.

**Figure 2:**
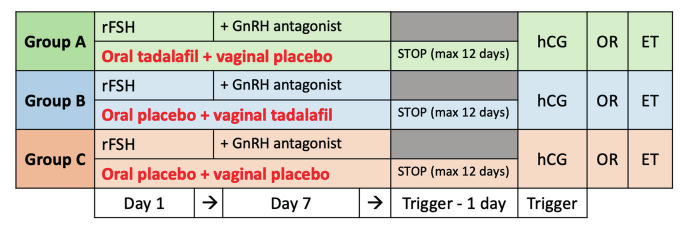
Study protocol. rFSH = recombinant follicular stimulating hormone, GnRH = gonadotropin-releasing hormone, hCG = human chorion gonadotrophin (ovulation trigger), OR = oocyte retrieval, ET = embryo transfer.

**Figure 3:**
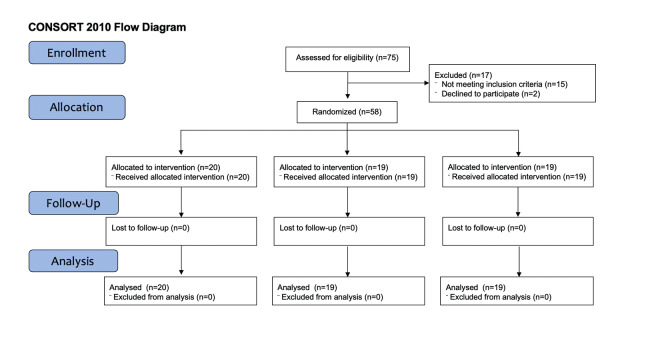
Flow diagram.

The study was carried out according to the criteria of the CONSORT statement (Schulz et al., 2010) (Figure 3).

### Treatment protocols

Treatment starts on the first day of the stimulation protocol and continues until the day of ovulation induction with a maximum of 12 days.

In all patients, a standard protocol (short protocol with gonadotropin-releasing hormone (GnRH) antagonist) was followed. Stimulation was performed with recombinant follicular stimulating hormone (rFSH). The starting dose of rFSH depends on the age of the patient and ovarian response in the previous cycle. Patients under 34 years old are starting with 150 units of rFSH per day, older patients, or patients with poor ovarian response in previous cycles are starting with 225 units per day.

Follow-up was performed using ultrasound measurements of the follicles, ultrasound assessments of the thickness of the endometrium and hormonal measurements by means of blood tests. All ultrasounds were performed by the same physician.

During the study there was no need for additional measurements or blood samples on top of the standard protocol for IVF treatment.

The data is stored according to protocol in the Jan Palfijn hospital. Data of patients who have left the study are stored.

### Outcome parameters

The primary outcome of this study is the thickness of the uterine lining. Endometrial thickness is determined by ultrasound as the distance between the transition lines between myometrium and endometrium, measured at the day of hCG triggering. Secondary outcomes are the rate of biochemical pregnancies and the rate of clinical pregnancies. Biochemical pregnancy is determined via blood sample with a value of hCG >= 25 IU/L 15 days after transfer of the embryo into the uterus. Clinical pregnancy is determined as an embryo with positive heart activity on ultrasound at 6 weeks amenorrhea.

### Statistical analysis

Comparison of treatment and control group characteristics was performed. The following characteristics were investigated: age, body mass index (BMI), day 2 - 3 follicle stimulating hormone (FSH), total dose of rFSH used in the stimulation, number of cumulus oocyte complexes (COC) obtained, number of oocytes in metaphase II (M2), number of two pronuclear embryos (2PN), number of cryo-conserved embryos and number of embryos transferred. These data were analysed using Student t-test.

Measurements of endometrial thickness and pregnancy tests were obtained from the patient file and processed anonymously. The statistical analysis was conducted by a one-way analysis of variance (ANOVA) test.

### Safety

In line with embryotoxic considerations, it was decided for safety reasons to avoid any contact between the active substance (tadalafil) and the embryo. The study medication was stopped on the day of ovulation induction, i.e., before follicle puncture, fertilisation, and transfer of the embryo. Tadalafil has a half-life of 17,5 hours (Gupta et al., 2005). At the moment of contact between the embryo and the maternal blood circulation, the medication is eliminated from the body.

## Results

Patient characteristics were similar in the three study groups (Table I). There was no significant difference between the groups in terms of age (p = 0.089) and in terms of BMI (p = 0.621). Basal FSH is also similar between the groups, indicating normal ovarian function. The dose of rFSH administered for ovarian stimulation in all groups was similar.

**Table I:** Patient characteristics.

		Group A	Group B	Group C	P-value
Age	Mean (SD)	32,45 (4,83)	29,21 (3,91)	30,32 (4,92)	0,089
	Median	31,5	28	30	
	Range	25 - 41	23 - 36	22 - 28	
BMI	Mean (SD)	24,33 (4,49)	23,32 (4,83)	24,69 (4,10)	0,621
	Median	23,42	22,30	25,21	
	Range	19,94 - 37,17	17,26 - 36,94	17,75 - 32,41	
FSH	Mean (SD)	8,12 (3,05)	7,77 (2,57)	9,30 (4,35)	0,356
	Median	8,39	8,07	8,5	
	Range	2,97 - 17,24	1,15 - 11,18	0,17 - 18,22	
DFSH	Mean (SD)	1955 (500)	2203 (567)	2143 (550)	0,311
	Median	1950	2025	2100	
	Range	1200 - 2700	1350 - 2775	1125 - 3375	
#COC	Mean (SD)	9,05 (6,08)	14,21 (4,34)	10,05 (6,55)	0,017
	Median	7	13	9	
	Range	1 - 25	5 - 22	3 - 27	
#M2	Mean (SD)	8,05 (6,18)	12,26 (3,93)	8,32 (6,48)	0,043
	Median	6	12	7	
	Range	1 - 25	3 - 21	1 - 26	
#2PN	Mean (SD)	6,00 (5,24)	8,16 (3,75)	4,84 (3,58)	0,061
	Median	5	8	4	
	Range	1 - 23	2 - 19	0 - 13	
Frozen embryo	Mean (SD)	1,60 (2,52)	2,37 (2,41)	0,84 (1,26)	0,100
	Median	0,5	2	0	
	Range	0-8	0-7	0-4	
TF	Mean (SD)	1,45 (0,51)	1,63 (0,50)	1,37 (0,60)	0,31
	Median	1	2	1	
	Range	1-2	1-2	0-2	

SD = standard deviation, BMI = body mass index, FSH = follicle stimulating hormone, DFSH = total dose of FSH used to stimulate before hCG trigger, #COC = number of cumulus oocyte complexes, #M2 = number of oocytes in metaphase 2, #2PN = number of embryos with 2 pronuclei, TF = number of embryos transferred.

The number of COC harvested after ovarian pickup was significantly different (p = 0.017) as was the number of M2 oocytes (p = 0.043). There was a significantly higher number of COC in group B (vaginal tadalafil), with a mean of 14.21, compared to group A (9.05) and group C (10.05). We see a similar effect in mature oocytes, with a mean of 12.26 mature oocytes in group B, 8.05 in group A and 8.32 in group C.

The difference is no longer significant when the number of oocytes with successful fertilization (2PN) is considered. The number of cryopreserved embryos is also not significantly different, but we can still retain a higher number of embryos in the treatment groups (group A and group B) compared to group C, which are on average 1.6 and 2.37 embryos respectively compared to only 0.84 embryos. In each transfer within this study, 1 or 2 embryos were transferred, according to the Belgian law. One patient had no embryo transfer.

No significant difference in endometrial thickness on the day of hCG triggering could be demonstrated between the three groups. The mean endometrial thickness in the study group treated with oral tadalafil (group A) was 10,47 mm (SD ± 2,34 mm), the group treated with vaginal tadalafil (group B) was 9,98 mm (SD ± 1,86 mm) and the control group (group C) was 10 mm (± 2,01 mm).

In terms of pregnancy rates, there is no significant difference between the three groups, but there seems to be a trend towards more biochemical and clinical pregnancies in group A.

Eight out of twenty patients (40%) achieved a biochemical pregnancy in group A, four out of nineteen in group B (21,05%) and two out of nineteen in group C (10,53%). Five out of twenty (25%) patients achieved a clinical pregnancy in group A, three out of nineteen (15,79%) in group B and two out of nineteen (10,53%) in group C.

The results were not significant; however, we see a trend towards more biochemical and clinical pregnancies in group A.

## Discussion

In our study we observed that there is no difference in mean endometrial thickness (p = 0,707), in biochemical pregnancies (p = 0.094) and in clinical pregnancies (p = 0,493) between the three groups (Table II).

**Table II:** Results.

		Group A	Group B	Group C	P-value
Endo	Mean (SD)	10,47 (2,35)	9,98 (1,86)	10 (2,01)	0,707
	Median	10,2	10,2	10,1	
	Range	6,5 - 14,7	5,9 - 13	5,4 - 13,4	
BP	Yes	40% (8/20)	21,05% (4/19)	10,53% (2/19)	0,094
	No	60% (12/20)	78,95% (15/19)	89,47% (17/19)	
CP	Yes	25% (5/20)	15,79% (3/19)	10,53% (2/19)	0,493
	No	75% (15/20)	84,21% (16/19)	89,47% (17/19)	

Endo = endometrial thickness in mm, BP = biochemical pregnancy, CP = clinical pregnancy.

In this study, we did not select our patient population based on thin endometrial thickness in previous cycles, due to the possibility of cycle-to-cycle variation within the same patient. Further research, exploring the effect of tadalafil in patients with persisting thin endometrium, might be indicated.

We found nine randomized controlled trials in the existing literature studying the use of PDE-5 inhibitors in fertility therapy. The main outcomes of these studies were endometrial thickness (four times), pregnancy rates (three times) and harvested oocytes (once). All studies were published between 2012 and 2017. The study populations ranged from 59 to 156.

Due to the large diversity within the studies (fertility treatments, dose, route of administration and treatment duration), it is not possible to draw one general conclusion about the role of the vasodilators in fertility treatment.

A Cochrane systematic review concerning the use of vasodilators for women undergoing fertility treatment (Gutarra-Vilchez et al., 2002) included 15 studies. The authors concluded there was insufficient evidence and further research is needed.

This study is the first to examine the effect of vaginal use of tadalafil.

In Table III you can find an overview of the existing literature.

**Table III:** Existing studies with PDE5 inhibitors in a table (RCTs only).

Trial	Fertility treatment	Treatment	Outcome		Patients	Control		Patients	p-value
Alieva et al. 2012	IVF	Sildenafil	Pregnancy	4,1%	29	No treatment	17,3%	30	
Deghani Firouzabadi et al. 2013	Frozen embryo transfer	Sildenafil (50mg)	ET	9,8 mm	40	Oestradiol valerate	8,0 mm	40	< 0,0001
Fahmy et al. 2015	OI (CC)	Vaginal sildenafil (25mg)	Pregnancy ET	65,7%	35		40%	35	0,031 0,014
Mangal and Mehirishi 2016	IUI	Vaginal sildenafil (4x25mg)	ET Pregnancy		50	Oestradiol valerate		50	0,14 0,042
Ataalla et al. 2016	IVF	Oral sildenafil (50mg)	Harvested oocytes	3,95 ± 1,395	30	Placebo	3,65 ± 1,137	30	0,681
Mekled et al. 2017	IVF	Oral sildenafil (4x25mg)	ET		40	Placebo		40	< 0,01
Fetih et al. 2017	CC	Vaginal sildenafil (2x50mg)	ET	9,3 ± 3,1 mm	42	Previous cycle	6,6 ± 1,4 mm	36	< 0,001
Mendez Lozano et al. 2015	IUI	Tadalafil (7x5mg)	ET	7,5 ± 2,1 mm			7,5 ± 2,1 mm		< 0,0002
Ammar et al. 2017	OI	Tadalafil (7x5mg)	Pregnancy		77			79	< 0,05

OI = ovulation induction, CC = clomiphene citrate, ET = endometrial thickness

Endometrial thickness might be a surrogate for the pregnancy outcome. The final goal of fertility therapy - and therefore of treatment with vasodilators - is pregnancy and birth, not the presence of a thick endometrium. However, a thin endometrium could lead to lower chances of success during implantation. There is no unequivocal evidence on this in literature.

The study by Groenewoud et al. (2018) in 463 patients could not demonstrate a relationship between increased endometrial thickness and pregnancy in freezing-thaw embryo transfer and concluded that there is therefore no predictive value for the chance of pregnancy.

Other studies do show a positive relationship. The study by Liu et al. (2018) analysed 44,477 embryo transfers and showed that for every millimetre less than 8 millimetres there is a decrease in the live birth rate in fresh and freeze-thaw cycles. The study by Gallos et al. (2018) analysed 25,767 fresh embryo transfers. This study showed a strong association between endometrial thickness and miscarriage/live birth. This group showed an optimal endometrial thickness of at least 10 millimetres (Gallos et al., 2018).

These findings show a relationship between endometrial thickness and pregnancy. The evidence is not unambiguous, but still strongly points towards a relationship.

Given the nature of this pilot study and the limited number of participants, it is not possible to draw general conclusions.

Endometrial thickness remains a surrogate outcome. A larger study with live birth rate or take- home baby is needed. Further investigation is needed to investigate which subgroups benefit most of the treatment with PDE-5 inhibitors.

## Conclusion

This study demonstrates the potential of long-acting PDE-5 inhibitors in fertility therapy. No significant difference was shown in endometrial thickness or incidence of biochemical pregnancies and clinical pregnancies between the different groups. However, a trend towards more pregnancies in the group treated with oral tadalafil is seen, based on better quality of the endometrium. More research in a larger study population is needed.
